# Effect of ultrasound on herpes simplex virus infection in cell culture

**DOI:** 10.1186/1743-422X-8-446

**Published:** 2011-09-22

**Authors:** Motoko Shintani, Gen Takahashi, Masakazu Hamada, Shyusuke Okunaga, Soichi Iwai, Yoshiaki Yura

**Affiliations:** 1 Department of Oral and Maxillofacial Surgery, Osaka University Graduate School of Dentistry, 1-8 Yamadaoka, Suita, Osaka 565-0871, Japan

## Abstract

**Background:**

Ultrasound has been shown to increase the efficiency of gene expression from retroviruses, adenoviruses and adeno-associated viruses. The effect of ultrasound to stimulate cell membrane permeabilization on infection with an oncolytic herpes simplex virus type 1 (HSV-1) was examined.

**Results:**

Vero monkey kidney cells were infected with HSV-1 and exposed to 1 MHz ultrasound after an adsorption period. The number of plaques was significantly greater than that of the untreated control. A combination of ultrasound and microbubbles further increased the plaque number. Similar results were obtained using a different type of HSV-1 and oral squamous cell carcinoma (SCC) cells. The appropriate intensity, duty cycle and time of ultrasound to increase the plaque number were 0.5 W/cm^2^, 20% duty cycle and 10 sec, respectively. Ultrasound with microbubbles at an intensity of 2.0 W/cm^2^, at 50% duty cycle, or for 40 sec reduced cell viability.

**Conclusion:**

These results indicate that ultrasound promotes the entry of oncolytic HSV-1 into cells. It may be useful to enhance the efficiency of HSV-1 infection in oncolytic virotherapy.

## Background

Oncolytic virotherapy is a novel way to destroy tumor cells using the cytopathic effect of non-virulent viruses [1-4]. Herpes simplex virus type 1 (HSV-1) vectors that lack the neurotoxic gene γ_1_34.5 have been developed and several vectors are under clinical trials [5-7]. A draw back of oncolytic virotherapy for solid tumors is the efficiency of infection. Many factors affect an infection. One important characteristic of the tumor microenvironment is the combination of a leaky vasculature and a lack of functional lymphatics, which can create increased interstitial fluid pressures [8,9]. Additional factors in the extracellular matrix of tumors can limit interstitial transport and as a result, further prevent the sufficient and uniform distribution of anti-cancer agents, especially large agents such as virus vectors [10,11]. The most reliable way to deliver oncolytic HSV-1 to solid tumors is direct inoculation, through a more efficient method of delivering HSV-1 to each tumor cell is required [7].

Ultrasound has been used diagnostically and therapeutically for decades, and its safety is well established [12,13]. Moreover, ultrasound as a means of stimulating cell membrane permeabilization, sonoporation, offers advantages over other technologies, primarily as a result of its relatively non-invasive nature [14,15]. It enhances the antitumor effect of chemotherapeutic agents and the delivery of plasmid DNA in vitro and in vivo [16,17]. The transiently increased permeability of the cell membrane is one of the mechanisms of ultrasound-enhanced chemotherapy. Usually, microbulles increased the efficiency of ultrasound exposure. Furthermore, ultrasound has been shown to increase the efficiency of gene expression from retroviruses, adenoviruses and adeno-associated viruses (AVVs) [18-21]. However, this method has not been applied to relatively large enveloped DNA viruses such as HSV-1. In the present study, we examined whether the infection of oncolytic HSV-1 is affected by ultrasound in the presence or absence of microbubbles.

## Materials and methods

### Cell culture and virus

The human oral squamous cell carcinoma (SCC) cell line SAS was obtained from the Japanese Collection of Research Bioresources (Tokyo, Japan). SAS cells were cultured in Dulbecco's modified Eagle's medium (DMEM) supplemented with 10% fetal bovine serum, 2 mM L-glutamine, 100 U/ml penicillin, and 100 μg/ml streptomycin and grown in an incubator at 37°C in a humidified atmosphere with 5% CO_2_. For Vero monkey kidney cells, Eagle's minimal essential medium containing 5% calf serum and 2 mM L-glutamine was used. The HSV-1 mutant R849 [22] and HF [23,24] were grown in semi-confluent Vero cell monolayers. Infected cells were subjected to three cycles of freezing and thawing and then centrifuged at 3,000 × g for 15 min at 4°C. The supernatant was kept at -80°C prior to use.

### Plaque assay

Cell monolayers were infected with virus serially diluted 10-fold. After an adsorption period of 60 min, unadsorbed viruses were removed by washing cell monolayers with phosphate-buffered saline (PBS) and then covered with medium containing 0.3% methylcellulose. They were incubated at 37°C in a humidified atmosphere with 5% CO_2 _for approximately 48 h. After the development of cytopathic effect, the cells were fixed in methanol, and stained by 1% crystal violet. The numbers of plaques was counted and plaque forming units (PFU)/ml were determined [25].

### Reagents and plates

As a microbubble, AS-0100 (Artison, Inola, OK) was used. This lipid-shelled ultrasound contrast agent filled with perfluorocarbon gas is composed of 9.8 × 10^8 ^microbubbles/ml, having an average diameter of 2.4 μm. A 24-well plate with a lumox ™ fluorocarbon film base was purchased from Greiner bio-one (Gottingen, Germany). The thickness of the gas-permeable film was 50 μm.

### Ultrasound

An ultrasound machine, Sonitron 2000 V (NEPAGENE Japan, Chiba, Japan), was used. The cells were grown onto alternate 24-well polystylene plates (Corning, NY) to prevent the exposure of neighboring cells [26]. Confluent cell monolayers were infected with 50 or 100 PFU of HSV-1. For sonoporation in the presence of microbubbles, virus in 90 μl of DMEM was mixed with microbubbles (9.8 × 10^6^/10 μl), after which the mixture was added to the cell cultures. The transducer was firmly fixed to a stand to avoid dislocation during exposure to ultrasound and the plates were placed on the head of the transducer with a diameter of 12 mm and contact was mediated using an ultrasound contact gel. After a period of viral adsorption, cells were exposed to ultrasound in the presence or absence of microbubbles. The ultrasound frequency was 1 MHz throughout the experiments. In an initial experiment, the ultrasound was adjusted to supply an intensity of 1.0 W/cm^2 ^(spatial average temporal peak) at a duty cycle of 10% (the pulse repetition rate, 100 Hz) for 20 sec. To determine optimal conditions, the parameters including intensity, duty cycle and period of exposure were changed. After the exposure, the mixture was removed and the cells were washed with PBS and subjected to a plaque formation assay. Experiments were divided into groups: virus infection only (Control); infection in the presence of microbubbles (MB); infection with ultrasound (US); infection with ultrasound in the presence of microbubbles (MB + US).

In the experiments to determine cell viability, dissociated cells (1 × 10^6^/900 μl) were mixed with microbubbles (9.8 × 10^7^/100 μl) and suspended in 48-well plates. After ultrasound, 1 × 10^4 ^cells were inoculated in 96-well plates.

### 3-(4,5-dimethylthiazol-2-yl)-2,5-diphenyl-tetrazolium bromide (MTT) assay

Cells inoculated in 96-well plates were incubated for 48 h. Thereafter, 10 μl of a 5 mg/ml MTT (Sigma, St.Louis, MO) solution was added to each well with 100 μl of medium and cells were incubated at 37°C for 4 h. After the addition of 100 μl of 0.04 N HCl in isopropanol, the plates were mixed thoroughly to dissolve the dark blue crystal and stood at room temperature overnight. The plates were read on a Benchmark Plus microplate spectrophotometer (Bio-Rad Laboratories, Hercules) with a reference wavelength of 630 nm and a test wavelength of 570 nm. Background absorbance at 630 nm was subtracted from the 570 nm reading. The values were divided by that of the control and the rate was calculated.

### Statistical analysis

All values were expressed as the mean ± SD. An Anova test was used to determine the significance of differences in multiple comparisons. The Mann-Whitney U-test was used to compare plaque numbers and cell viability between each treatment group and the control, and between the ultrasound group and ultrasound with microbubble group. These statistical analyses were performed using the software Statcel2, version 2 (OMS, Tokyo, Japan). A value of P < 0.05 was considered to be statistically significant.

## Results

### Effect of microbubbles on the infectivity of HSV-1

**Figure 1:**
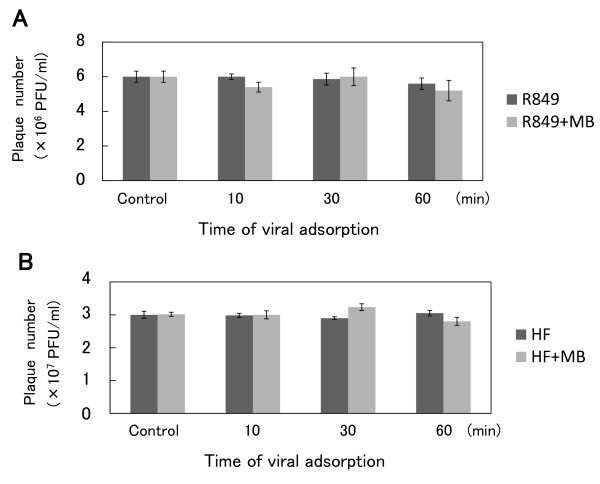
**Effect of microbubbles on the infectivity of HSV-1**. (A) Microbubbles (9.8 × 10^7^/100 μl) were mixed with viral solution (1 × 10^7 ^PFU of R849/900 μl). These mixtures were placed at 4°C. After incubation for 10, 30, and 60 min, the mixtures were subjected to plaque assays on Vero cell monolayers. (B) HSV-1 strain HF was also tested for sensitivity to microbubbles. Microbubbles and 3 × 10^7 ^PFU of HF were mixed as described for R849.

Whether microbubbles, AS-0100, could affect the infectivity of HSV-1 R849 was examined. Microbubbles (9.8 × 10^7^/100 μl) were mixed with viral solution (1 × 10^7 ^PFU/900 μl). These mixtures were placed at 4°C. After an incubation for 10, 30, or 60 min, the mixtures were serially diluted with cold PBS and subjected to plaque assays on Vero cell monolayers. It was found that the plaque numbers in the mixtures incubated at 4°C for 60 min were not significantly decreased (Figure 1). When 3 × 10^7 ^PFU of another HSV-1 strain, HF, were mixed with microbubbles, no significant reduction in plaque numbers was observed.

### Effect of ultrasound on the plaque formation by R849 in Vero cell monolayers

**Figure 2:**
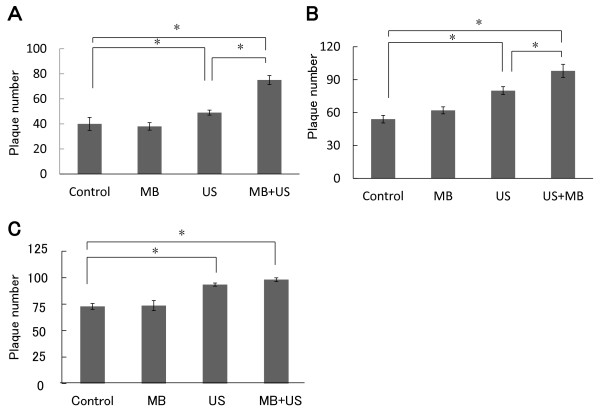
**Effect of ultrasound on the plaque-forming ability of R849 in Vero cell monolayers**. Vero cells were inoculated with 100 PFU of R849 and incubated at 37°C for 10 (A), 30 (B), or 60 (C) min. Thereafter, cell monolayers were exposed to ultrasound in the presence (M + U) or absence (U) of microbubbles, washed with PBS and covered with medium containing methylcellulose. After further incubation for 48 h, the number of plaques was determined. Infected cells were also incubated in the presence of micrbubbles (M) or left untreated (Control) during the adsorption period and used for plaque formation. Data are means ± SDs of three determinations. *P < 0.05.

Since viruses attach to the cell surface and enter into cells in a time-dependent manner, the number of plaques increases on prolonging the adsorption period. To determine the effect of ultrasound on viral entry, Vero cells were inoculated with 100 PFU of R849 with or without microbubbles. After incubation at 37°C, infected cells were exposed to ultrasound and then unadsorbed viruses were removed and cells were incubated for plaque formation. The intensity, duty cycle and exposure time were 1.0 W/cm^2^, 10% and 20 sec, respectively. In the cultures exposed to ultrasound after 10, 30 or 60 min of adsorption, mean plaque numbers were increased 1.2-fold, 1.5-fold and 1.3-fold as compared with the control, respectively (Figure 2). In the presence of microbubbles, the increases by ultrasound after 10, 30 or 60 min were 1.9-fold, 1.8-fold and 1.4-fold as compared with the control, respectively. There was a significant difference between the control group and ultrasound group (P < 0.05) or ultrasound with microbubbule group (P < 0.01). A significant difference (P < 0.05) was also observed between the ultrasound group and ultrasound with microbubble group. There was no increase in the experiment with microbubbles only.

### Effect of ultrasound on the plaque formation by HF in Vero cells and by R849 in oral SCC cells

**Figure 3:**
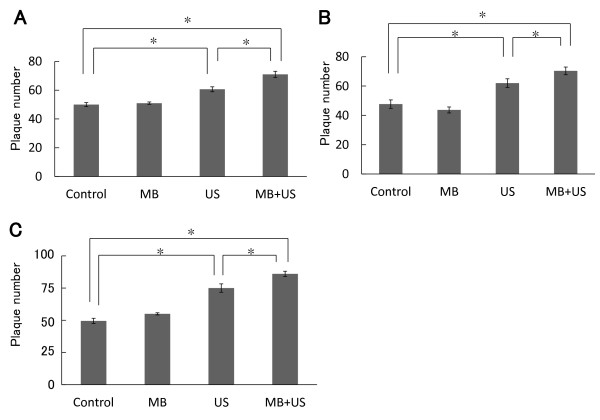
**Effect of ultrasound on the formation of plaques by HF in Vero cells, and by R849 in SAS cells**. (A) Vero cells were inoculated with 50 PFU of a fusogenic HSV-1 mutant HF and cultured at 37°C for 30 min. Thereafter, cell monolayers were exposed to ultrasound in the presence or absence of microbubbles as described in Figure 2. After further incubation, the number of plaques was determined. (B) SAS cells were inoculated with 100 PFU of R849 and cultured for 30 min. They were treated as described in (A). (C) Vero cells grown in lumox ™ multiwell plates were inoculated with 100 PFU of R849 and cultured for 30 min. They were treated as described in (A). Data are means ± SDs of three determinations. *P < 0.05.

The HSV-1 mutant HF was fusogenic and produced large plaques in Vero cells. The inoculation was reduced to 50 PFU. After 30 min of adsorption, cells were exposed to ultrasound in the presence or absence of microbubbles. The mean number of plaques was significantly (P < 0.05) greater than that of the control (Figure 3A).

The effect of ultrasound was also examined in human oral SCC SAS cells. Cell monolayers were inoculated with 100 PFU of R849. After 30 min of adsorption, cells were exposed to ultrasound as described for Vero cell monolayers. The increases in plaque numbers caused by ultrasound or ultrasound with microbubbles were 1.5- fold and 1.8-fold as compared with the control (Figure 3B).

The transparent base of the lumox ™ multiwall is made of an ultra thin gas-permeable film with low autofluorescence [27]. Vero cells were grown on lumox ™ multiwall plates and infected with 100 PFU of R849. When cells were exposed to ultrasound after 30 min of adsorption in the absence or presence of microbubbles, the mean numbers of plaque were increased 1.5-fold and 1.7-fold as compared with the control (Figure 3C). The difference between the control group and ultrasound or ultrasound with microbubble group was significant (P < 0.05). In the presence of microbubbles, the number of plaques was further increased.

### Effect of ultrasound intensity, duty cycle, and exposure time on the formation of plaques by HSV- 1

**Figure 4:**
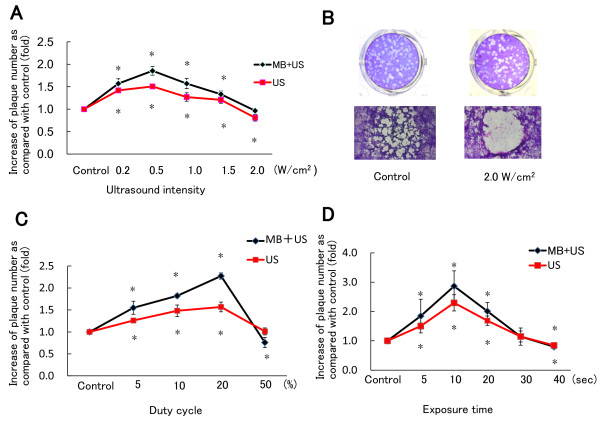
**Effect of ultrasound intensity, duty cycle, and exposure time on the formation of plaques by HSV-1**. (A) Vero cells were inoculated with 100 PFU of R849 and incubated for 30 min. Thereafter, cell monolayers were exposed to ultrasound in the presence or absence of microbubbles. The duty cycle and exposure time were fixed to 10% and 20 sec, and the ultrasound intensity was changed from 0.5 to 2.0 W/cm^2^, according to Sonitron intensity dial settings. After further incubation, the number of plaques was determined and the rate relative to the control was calculated. (B) The morphology of the control and cells exposed to ultrasound at an output intensity of 2.0 W/cm^2 ^in the presence of microbubbles. (C) R849-infected Vero cells were exposed to ultrasound and the number of plaques was determined as described in (A). The output intensity and exposure time were fixed to 0.5 W/cm^2 ^and 20 sec and the duty cycle was changed from 5 to 50%. (D) R849-infected Vero cells were exposed to ultrasound and the number of plaques was determined as described in (A). The output intensity and duty cycle were fixed to 0.5 W/cm^2 ^and 20% and the duty cycle was changed from 5 to 40 sec. Data are means ± SDs of three determinations. *P < 0.05 vs. control.

In the above experiments, cells were exposed to ultrasound under conditions used for chemotherapeutic drugs and plasmid DNA [28,29]. To determine the optimal conditions for HSV-1 infection, experiments were undertaken at 30 min after infection, using Vero cells and R849. First, the duty cycle and exposure time were fixed to 10% and 20 sec, and the ultrasound intensity was changed from 0.5 to 2.0 W/cm^2^. The mean plaque number reached a maximal level at 0.5 W/cm^2 ^(Figure 4A). Microbubbles further increased the plaque number and the difference between the ultrasound group and ultrasound with microbubble group was significant (P < 0.05). However, at 2.0 W/cm^2^, the plaque number was decreased as compared with the control. Cell shrinkage and enlarged intercellular spaces were observed (Figure 4B). On microscopic observation of plaques, control cells remained attached to the plate, whereas most cells exposed to ultrasound were lost. Second, the output intensity and exposure time were fixed to 0.5 W/cm^2 ^and 20 sec, and the duty cycle was changed. A duty cycle ranging from 5% to 20% increased the plaque number, with the maximum effect achieved at 20% (Figure 4C). Microbubbles further increased plaque numbers. At a duty cycle of 50%, however, the plaque number decreased in the presence of microbubbles. Third, the output intensity and duty cycle were fixed at 0.5 W/cm^2 ^and 20% and the exposure time was changed from 5 to 40 sec. The plaque numbers reached a maximal level at 10 sec (Figure 4D). There was a significant (P < 0.05) difference between the ultrasound group and ultrasound with microbubble group.

### Effect of ultrasound and microbubbles on the infectivity of HSV-1

To determine whether ultrasound and microbubbles could affect the infectivity of HSV-1 R849, viral solution (8 × 10^4 ^PFU/180 μl) was mixed with microbubbles (1.9 × 10^6^/20 μl) in a 24-well plate and then exposed to ultrasound irradiation. The intensity, duty cycle and exposure time were 0.5 W/cm^2^, 20% and 10 sec, respectively. In unirradiated control, PBS was added instead of microbubbles. When the mixtures were subjected to plaque assay, the mean titers in unirradiated control, ultrasound only, and ultrasound with microbubbles were 3.5 × 10^5 ^PFU/ml, 3.2 × 10^5 ^PFU/ml, and 3.4 × 10^5 ^PFU/ml, respectively. There was no reduction of the infectivity of R849 by ultrasound in the presence or absence of microbubbles.

### Effect of ultrasound on cell viability

**Figure 5:**
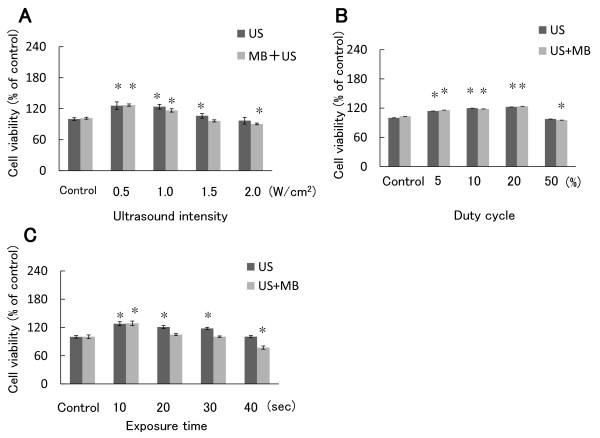
**Effect of ultrasound on the viability of SAS cells**. (A) SAS cell suspension was exposed to ultrasound in the presence or absence of microbubbles. Cells were incubated in 96-well plates for 48 h and cell viability was measured by MTT assay. The duty cycle and exposure time were fixed to 10% and 20 sec and output intensity was changed from 0.5 to 2.0 W/cm^2^, according to Sonitron intensity dial settings. (B) The output intensity and exposure time were fixed to 0.5 W/cm^2 ^and 20 sec and duty cycle output was changed from 5 to 50%. (C) The output intensity and duty cycle were fixed to 0.5 W/cm^2 ^and 10% and exposure time was changed from 10 to 40 sec. The values were divided by that of the control and the percentage was calculated. Data are means ± SDs of three determinations. *P < 0.05 vs. control.

To determine the effect of ultrasound on cell viability, uninfected SAS cells were exposed to ultrasound in cell suspensions and cell viability was measured by MTT assay. First, the duty cycle and exposure time were fixed to 10% and 20 sec, and output intensity was changed from 0.5 to 2.0 W/cm^2^. From 0.5 to 1.5 W/cm^2^, cell viability was slightly increased, but it was decreased to 89% of the control at an intensity of 2.0 W/cm^2 ^in the presence of microbubbles (Figure 5A). Second, when the duty cycle of ultrasound was changed, cell viability decreased to 92% of the control at a duty cycle of 50% in the presence of microbubbles (Figure 5B). Third, when exposure time for ultrasound was changed, cell viability decreased to 81% of the control after exposure for 40 sec in the presence of microbubbles (Figure 5C). There were significant differences (P < 0.05) between the control group and ultrasound with microbubble group.

## Discussion

Ultrasound has been studied extensively as a delivery method for chemotherapeutic agents and plasmid DNA because of its low invasiveness, but studies using infectious viruses are limited. For transduction of the green fluorescence protein (GFP) gene, Li et al. [30] tested three ultrasound contrast agents, Albunex (an albumin-shelled contrast agent composed of air-filled microbubbles), Optison (a perfluorocarbon-filled contrast agent) and Levovist (a galactose-based and air-filled contrast agent). They indicated that the mean number of GFP-transfected cells was approximately 8-fold greater in the presence of Optison than Albunex or Levovit. In the present study, we used AS-0100 composed of a lipid shell containing perfluorocarbon gas. First, we tested the sensitivity of HSV-1 to the microbubbles but found no apparent reduction in plaque-forming ability after incubation for 60 min. Therefore, the microbubbles did not affect the infectivity of HSV-1.

Howard et al. [19] reported that an adenovirus carrying the GFP marker gene could be transduced efficiently into human prostate, lung and melanoma cells via ultrasound exposure in the presence of microbubbles, but not with ultrasound only. Ultrasound with microbubbles also enhanced the expression of GFP encoded in AAV [21]. Although these studies demonstrated the efficiency of gene expression by ultrasound, the effect on the entry of the virus was not studied. In the present study, cells were inoculated with 100 PFU of HSV-1 and exposed to ultrasound after different time intervals. Indeed, when cells were exposed to ultrasound after a period of viral adsorption, the plaque number significantly increased. The effect of ultrasound was not confined to a specific strain of virus or cell line. Furthermore, the infectivity of R849 was not affected by ultrasound irradiation in the presence of absence of microbubbles. Thus, we concluded that ultrasound could accelerate the entry of oncolytic HSV-1 into oral SCC cells. Even in experiments using a lumox ™ multiwell plate with a thin fluorocarbon film, a similar enhancement of plaque-forming activity was observed. The findings obtained with polystylene plates may be equivalent to those for the lumox ™ plate.

Li et al. [21] infected to human retinal pigmented epithelial cells with AAV under ultrasound conditions, namely, 1.0 W/cm^2^, a duty cycle of 20% and an exposure period of 20 sec and found an increase in the GFP-positive rate from 17 to 32%. We performed experiments in a variety of conditions and found that an intensity of 0.5 W/cm^2^, duty cycle of 20% and exposure time of 10 sec were the optimal parameters to attain a maximal number of plaques. It is essential to optimize parameters for the efficient transduction of HSV-1. Recently, Kopechek et al. [31] calculated the acoustic output in a cell-well plate, using Sonitron 1000 and 2000 and 3 types of transducer. Although the experimental conditions were not identical to ours, their results must be useful to estimate the peak-to-peak acoustic pressure at Sonitron intensity settings. Li et al. [21] also examined the effect of the ratios of microbubbles to cells and found that the percentage of transfection efficiency in 50:1 group was increased as compared with 20:1 group. In the present study, we used microbubbles following the recommendation by the manufacturer, so that the ratio of microbubbles to cells was approximately 20:1 for HSV-1 infection. Although the optimal ratio remained to be clarified, it can be stated that the amounts of microbubbles used were sufficient to promote the entry of HSV-1 into epithelial cells.

In cancer therapy, the cytotoxicity of ultrasound would concomitantly have cell-killing effects [32,33]. However, if cell viability was impaired by sonoporation, the replication and antitumor ability of virus would be blunted. We found that ultrasound exposure at 2 W/cm^2 ^induced changes in cell morphology and decreased cell viability. Duty cycle and exposure time also affected the cell viability. Less invasive conditions are required for efficient HSV-1 infection. In the study of plaque assay, confluent monolayers are required during virus adsorption. When confluent monolayers were exposed to ultrasound, further growth of cells is not expected and only severe cell damage can be detectable by MTT assay. Thus, we decided to use cell suspension and plated the cells after ultrasound for MTT assay. The re-plated cells proliferated at a similar rate as unirradiated cells, if the intensity, duty cycle and time were within a range. This is consistent with the result of plaque assay in which monolayers were used and no cytotoxic alteration of cell morphology occurred except for the condition at high ultrasound intensity (Figure 4B). Although the difference in exposure conditions including cell adhesion and well size may yield distinctly different results, it is unlikely that ultrasound induces lytic changes of the cell membrane to facilitate the virus entry. If this is the case, the number and size of plaques formed in Vero and SAS cell monolayers would be reduced, because plaque formation is dependent on the viability of cells.

In HSV-1 infection, as an initial step, glycoproteins gB and gC bind to heparin sulfate proteoglycans on the cell surface, attaching the virus to the host cell. Once the viral and host cell membranes are brought close to each other, glycoprotein gD can associate with any of a number of cell receptors including herpesvirus entry mediator (HVEM), nectin-1 and 3-O-sulfated heparin sulfate to trigger fusion. However, at least three pathways are implicated in HSV-1's entry into different types of susceptible cells: direct fusion with the host cell membrane, endocytosis followed by fusion with an acidic endosome, and endocytosis followed by fusion with a neutral endosome [34-36]. Taylor et al. [20] introduced envelope-deficient retroviral vectors into human rhabdomyosarcoma cells, suggesting that an unenveloped-retrovirus could enter the cells through ultrasound-generated pores of the plasma membrane. We found efficient entry by ultrasound, and a further increase with microbubbles. It is possible that the ultrasound-induced formation of pores contributes to the entry of HSV-1. If so, a similar increase in plaque number should be observed, irrespective of the adsorption period. However, ultrasound produced more plaques after 30 min of adsorption than 10 min of adsorption. For example, the increases in cultures treated after 10 or 30 min of adsorption were 10 and 27 PFU, respectively (Figure 2a, b). Thus, another mechanism would be present. In this regard, Hernot and Klibanov [37] proposed the involvement of active transport mechanisms, such as endocytosis and phagocytosis, in the uptake of microbubbles under ultrasound. In the entry of HSV-1 that attaches to the cell surface, endocytosis may be promoted by ultrasound. These in vitro data must be useful to demonstrate the efficacy of sonoporation in introducing R849 and HF into oral SCC xenografts in nude mice.

## Conclusion

The exposure of Vero cells and oral SCC cells to ultrasound can accelerate the process of HSV-1 infection. Microbubbles further enhanced the effect of ultrasound. Ultrasound may be useful to enhance the efficiency of HSV-1 infection in oncolytic virotherapy for oral SCC.

## Competing interests

The authors declare that they have no competing interests.

## Authors' contributions

MS performed major part of the experiments, GT contributed to the design and conduction of ultrasound studies, MH performed statistical analysis, SI participated in drafting the manuscript, SO performed experiments for virus stability and YY was responsible for the project and for the preparation of the manuscript.
